# Cerebrospinal-fluid exposure of efavirenz and its major metabolites when dosed at 400 and 600 mg once daily; a randomized controlled trial

**DOI:** 10.7448/IAS.17.4.19541

**Published:** 2014-11-02

**Authors:** Alan Winston, Rebekah and Puls, group Encore CSF Sub-study

**Affiliations:** 1 Department of Medicine, Imperial College London, London, UK; 2 Kirby Institute, TVRP, Sydney, Australia

## Abstract

**Introduction:**

The optimal penetration of antiretroviral agents into central nervous system (CNS) may be a balance between providing adequate drug exposure to inhibit HIV-replication whilst avoiding concentrations associated with toxicities.

**Methods:**

Cerebrospinal-fluid (CSF) exposure of efavirenz and metabolites 7-hydroxy (7OH-) and 8OH-efavirenz were assessed after at least 12 weeks of antiretroviral therapy in HIV-infected subjects randomized to commence antiretroviral regimens containing efavirenz at either 400 mg or 600 mg once daily. Clinical, pharmacokinetic and pharmacogenomic factors associated with CSF efavirenz and its metabolite concentrations were assessed.

**Results:**

Of 28 subjects who completed all study procedures (14/14 on efavirenz 400 mg/600 mg), CSF HIV RNA was below 20 copies/mL in all at the time of examination. Concentrations of efavirenz and 7OH-efavirenz in the CSF were slightly lower when dosed at 400 mg versus 600 mg, although this was not statistically significant. A different trend was observed regarding 8OH-efavirenz concentrations where CSF exposure was slightly increased in the 400 mg efavirenz arm (see Table 1). Efavirenz concentration in the CSF was above 0.51 ng/mL (proposed CSF IC50 for WT virus) in all subjects and 8OH-efavirenz concentration in the CSF was above 3.3 ng/mL (a proposed toxicity threshold, *reference*) in 11/14 and 7/14 subjects randomized to the 400 mg and 600 mg doses of efavirenz, respectively. Whilst CSF efavirenz concentration was significantly associated with plasma concentration (P<0.001) and CYP2B6 genotype (CSF efavirenz GG to GT/TT GM ratio 0.56, 90% CI 0.42–0.74), CSF 8OH-efavirenz concentration was not (P=0.242 for association with plasma concentration and CSF 8OH-efavirenz GG to GT/TT GM ratio 1.52, 90% CI 0.97–2.36). Lastly, CSF 8OH-efavirenz concentration was associated with efavirenz symptom questionnaire results at one year (Spearman's correlation 0.13, P=0.05).

**Conclusions:**

With both doses of efavirenz studied, CSF concentrations were considered adequate to inhibit HIV-replication, although concentrations of 8OH-efavirenz were greater than that reportedly associated with neuronal toxicity. CSF exposure of 8OH-efavirenz was not dependent on plasma exposure and we postulate may be subject to saturable pharmacokinetic effects.

**Table 1:** CSF geometric means and ratios of efavirenz and metabolite concentrations by randomized arm (Ratios are 400/600 mg)

		Geometric means (ng/mL)		
				
Parameter	Arm	Mean	90% CI (lower)	90% CI (upper)
CSF efavirenz	400 mg	16.4	13.0	20.7
	600 mg	19.5	15.1	25.1
	ratio	0.84	0.61	1.18
CSF 7OH-efavirenz	400 mg	0.62	0.41	0.93
	600 mg	0.63	0.40	0.99
	ratio	0.98	0.55	1.79
CSF 8-OH-efavirenz	400 mg	5.08	4.0	6.44
	600 mg	3.08	2.13	4.43
	ratio	1.65	1.09	2.5
